# Draft Genome Sequence of the Lichenized Fungus Bacidia gigantensis

**DOI:** 10.1128/MRA.00686-21

**Published:** 2021-11-04

**Authors:** Jessica L. Allen, Steven J. M. Jones, R. Troy McMullin

**Affiliations:** a Biology Department, Eastern Washington University, Cheney, Washington, USA; b Canada’s Michael Smith Genome Sciences Centre, British Columbia Cancer Agency, Vancouver, British Columbia, Canada; c Research and Collections, Canadian Museum of Nature, Ottawa, Ontario, Canada; Vanderbilt University

## Abstract

The draft genome sequence of Bacidia gigantensis, a lichenized fungus in the order Lecanorales, was sequenced directly from a herbarium specimen collected from the type locality at Sleeping Giant Provincial Park in Ontario, Canada. Using long-read sequencing on the Oxford Nanopore PromethION platform, we assembled a nearly complete genome sequence.

## ANNOUNCEMENT

We sequenced the genome of Bacidia gigantensis, a crustose, epiphytic, lichenized fungus in the family Ramalinaceae. The species was recently described as new to science from Sleeping Giant Provincial Park along the north shore of Lake Superior in Ontario, Canada (1). A whole lichen specimen was collected at the type locality (1), and DNA was extracted from whole thallus material. The tissue was disrupted using metal bead-based homogenization; proteins were pelleted via centrifugation and the DNA-containing supernatant drawn off. Then, multiple ethanol washes were conducted over a silica column. All steps followed a protocol similar to those for commercially available kits (2). Bead cleanup at a 0.6:1 bead-to-sample ratio was first performed to remove small DNA fragments using PCR Clean DX C-1003-450 (Aline Biosciences, Woburn, MA, USA). The quality of the genomic DNA (gDNA) was assessed using pulse-field gel electrophoresis (PFGE) with 1% agarose gel by running with 0.5× Tris-borate-EDTA (TBE) on a CHEF pulse field gel apparatus (Bio-Rad, Hercules, CA, USA) for 22 h. The library construction and sequencing were conducted following Oxford Nanopore’s genomic DNA by ligation (SQK-LSK109) protocol. Briefly, 1.14 μg of genomic DNA was formalin-fixed, paraffin-embedded (FFPE) repaired, end-prepped, and dA-tailed using the NEBNext FFPE DNA repair mix (M6630) and the NEBNext Ultra II end prep/dA-tailing module (E7546). NEBNext quick ligase (E6056S) was used to ligate the Oxford Nanopore adapters. A final size selection using a 0.4:1 bead-to-library ratio was performed to select against smaller molecules. Then, 380 ng of the final library was loaded onto a PromethION 24 instrument using the R9.4.1 pore flow cell and v19.06.9 software (MinKNOW graphical user interface [GUI] v4.0.23). Sequencing was run for 72 h. Base calling was performed using Fast-Bonito v0.2.2 (https://github.com/nanoporetech/bonito). The *N*_50_ value of the reads was 26 kb. A total of 14,958,030 reads containing 32 Gbp were generated.

The reads were corrected using Canu v2.0 (3); they were then assembled using Flye v2.8.2 with “--pacbio-hifi” mode, the option “--hifi-error 0.006,” and using the purge_haplotigs tool to remove duplicated disjointigs between the “consensus” and “repeat” stages of the Flye assembly pipeline (4, 5). The assembly was polished by mapping all of the reads to the assembly using Minimap2 v2.17, then implementing one round of Racon v1.4.17 polishing and one round of Medaka v1.2 polishing (6) (https://github.com/nanoporetech/medaka). To remove nontarget organism sequences from the assembly, we used a Diamond v0.9.32 blastx search against the NCBI nonredundant protein database to assign the taxa to each scaffold, then built an annotated GC depth plot using the BITAT python script from McKenzie et al. (7) (https://github.com/biorover/LethariaGenomes). We then kept only scaffolds with 70× to 80× coverage and a GC content below 0.6 that were annotated as Ascomycota, as was the *Bacidia gigantensis* genome. We used QUAST v5.0.2 to evaluate the final metrics for the assembly. The assembly was highly contiguous and comprised 24 scaffolds, with a total length of 33.12 Mb, with an *N*_50_ value of 18.1 Mb and an *L*_50_ value of 8 Mb (8). The genome sequence was also highly complete, with a BUSCO v3.1 homology search against the Pezizomycotina database, resulting in 92.7% complete single-copy genes, 0.5% duplicate single-copy genes, 2.6% incomplete genes, and 4.6% missing genes (9).

We used Funannotate v1.8.7 for gene prediction in the nuclear genome using all default evidence-guided and *ab initio* predictors (https://github.com/nextgenusfs/funannotate). We first masked the repeat regions, which comprised 7.69% of the genome. Protein evidence was derived from the Joint Genome Institute database for the following accessions: Cladonia grayi Cgr/DA2myc/ss v2.0, Lobaria pulmonaria Scotland reference genome v1.0, Usnea florida ATCC 18376 v1.0, and Xanthoria parietina 46-1-SA22 v1.1. Aspergillus fumigatus was selected as the seed species for BUSCO searches. A total of 14,923 valid annotations were compiled, and all genome statistics are summarized in Table 1.

**TABLE 1:** Summary statistics for the *Bacidia gigantensis* genome assembly and annotation

Characteristic^*a*^	Value
Total assembly length (Mb)	33.12
No. of scaffolds	24
*N*_50_ (Mb)	18.1
*L*_50_	8
No. of complete single-copy BUSCOs (%)	1,186 (92.7)
No. of duplicate single-copy BUSCOs (%)	1 (0.5)
No. of incomplete BUSCOs (%)	35 (2.6)
No. of missing BUSCOs (%)	35 (4.6)
% repeat	7.69
%GC	44.67
No. of tRNAs	51
No. of Phobius secretome genes	931
No. of Phobius transmembrane proteins	1,798
No. of antiSMASH biosynthetic gene clusters	35
No. of antiSMASH biosynthetic enzymes	78
No. of antiSMASH smCOGs	103
No. of CAZYmes	205
Total no. of annotations	14,923
NCBI BioProject accession no.	PRJNA748063
Sequence Read Archive accession no.	SRX11510989
GenBank assembly accession no.	GCA_019456465.1

a BUSCOs, benchmarking universal single-copy orthologs; smCOGs, secondary metabolism clusters of orthologous groups; CAZYmes, carbohydrate-active enzymes.

### Data availability.

All data are available under the National Center for Biotechnology Information BioProject accession number PRJNA748063. The raw reads are available under the Sequence Read Archive accession number SRX11510989; the assembled nuclear genome sequence is available under the GenBank assembly accession number GCA_019456465.1.
